# Supplementary material for: Development of a novel EV-A71 monoclonal antibody for monitoring vaccine potency
Source: PLoS Negl Trop Dis. 2025 Jun 3;19(6):e0013127. doi: 10.1371/journal.pntd.0013127 (PMC12157779; doi:10.1371/journal.pntd.0013127)
Supplement: S1 Table — (XLSX) [file pntd.0013127.s001.docx]

| Primer | Sequence (5’-3’) | Function |
| --- | --- | --- |
| 141-Forward | ACAGCCTCTATTCAGGGAGATAGGGTGGCAGATGTGAT | PCR |
| 141-Reverse | CTGGCCGAACTTTCCAAGGGTAGTAATGGCGGTACGACTA | PCR |
| Clone 92-forward | GGAAAGTTCGGCCAGCAATCTGGGGCCATCTA | PCR |
| Clone 92 reverse | CTGAATAGAGGCTGTCTGTAATATGTGACTAGT | PCR |
| BamHI-1460 F | TCGATGCTGGGATCCCTATATCGCA | PCR |
| AflII-3672 R | TGTTTGGCACCTTAAGATGCCTCCGC | PCR |
| 3672 AflII-F | GGA GGC ATC TTA AGG TGC CAA CAT | PCR |
| Uni-F-AfIII-R | TTTATACTTAAGGTTTTTTTTTTTTTTTTTTTTTTTTTTTTTTTGCTATTCTGGTTATAACAAATT | PCR |
| NotI 1-F | ATAAGAATGCGGCCGCTTAAAACAGCCTGTGGGTTGCACC | PCR |
| BamHI 1460-R | CGGGATCCCAGCATCGAGCACATACGG | PCR |
| EV71-VP3-S64R-F | CCCACCAATGCTACTAGGTTGATGGAAAGACTACG | PCR |
| EV71-VP3-S64R-R | CGTAGTCTTTCCATCAACCTAGTAGCATTGGTGGG | PCR |
| VP3-L101L-F | ATGCTAGGCCAGTTGTGTGGATAC | PCR |
| VP3-L101L-R | TGGCCTAGCATCGTTGACTGCCAT | PCR |
| VP3-Y202C-F | CTGGTATCAAACAAATTG CGT GG | PCR |
| VP3-Y202C-R | CCC CTA TTG GGA CCA CGC AAT T | PCR |
| T7P | TAATACGACTCACTATAGGG | Sequencing |
| EV71-577-F | CTCACATTGGCTGCTTATGG | Sequencing |
| EV71-434F-VP2 | ACAGTCACCCTCCTTAC | Sequencing |
| EV71-426F-VP3 | ACCGGGCTACAGCAATG | Sequencing |
| EV71-472F-VP1 | CCCTGGTGCTCCTAAAC | Sequencing |
| EV71-3679-F | TGCCAACATGGTGTAGTTGG | Sequencing |
| EV71-984F-P2 | ACCTCGCTCATTTCTGTC | Sequencing |
| EV71-1716F-P2 | AGCACTATTCCAAGGCCCG | Sequencing |
| EV71-713F-P3 | GCCAACACATAGGACCATGATG | Sequencing |
| EV3-B5-2507-R | TGGGTGAGTGCCCTACTCACAC | Sequencing |
| EV71-1423F-P3 | ACCTCAGAATGACCTTTGGGC | Sequencing |
| EV71-7074-F | GTTCTTGCCAGACCACCAAT | Sequencing |
