# Supplementary figures and images for: Development of a novel EV-A71 monoclonal antibody for monitoring vaccine potency
Source: PLoS Negl Trop Dis. 2025 Jun 3;19(6):e0013127. doi: 10.1371/journal.pntd.0013127 (PMC12157779; doi:10.1371/journal.pntd.0013127)

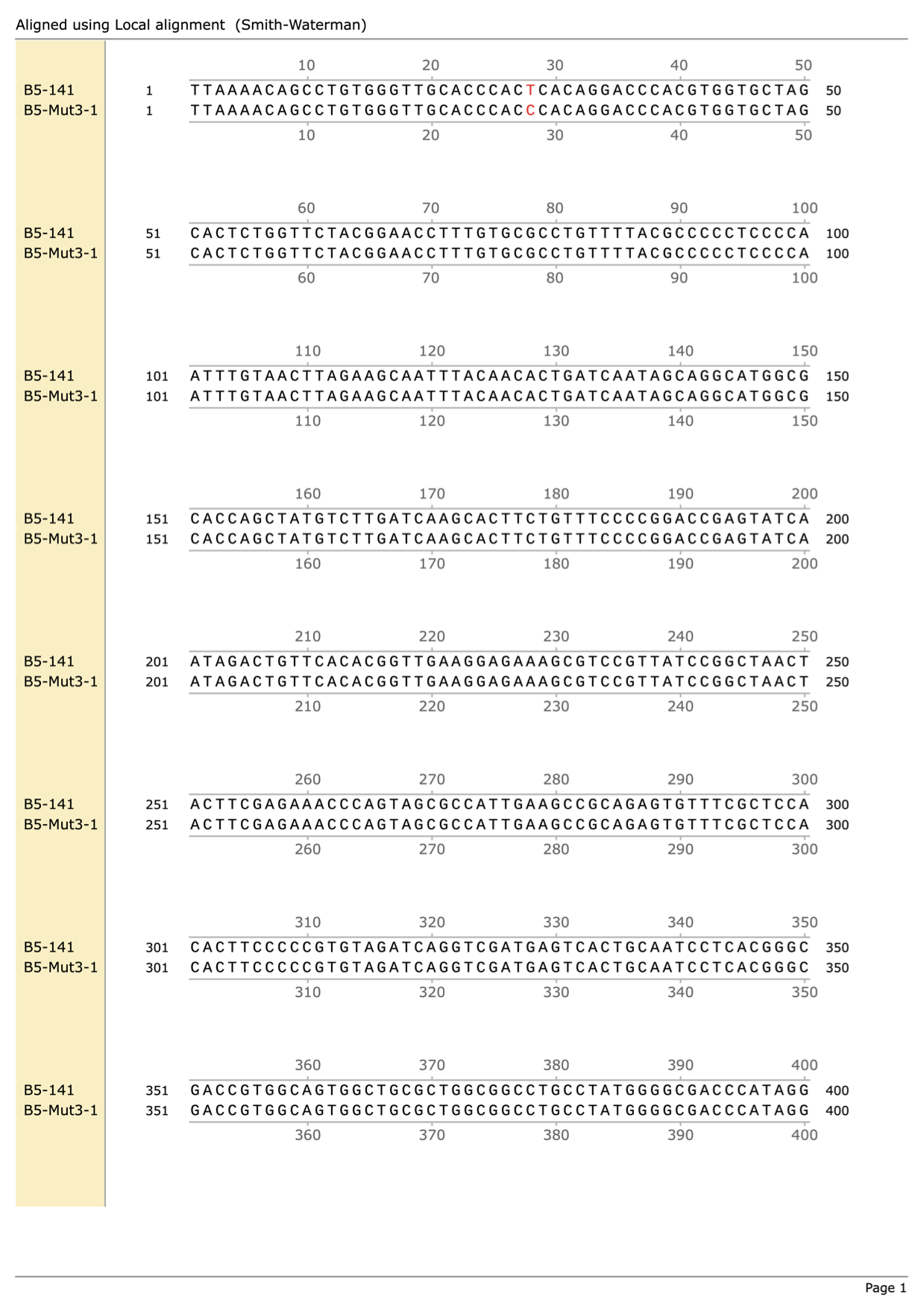

Supplement: S1 Fig — Consensus nucleotides are depicted in black, while non-consensus nucleotides are highlighted in red. (TIFF) [file pntd.0013127.s003.tiff]
