# Supplementary material for: Development of a novel EV-A71 monoclonal antibody for monitoring vaccine potency
Source: PLoS Negl Trop Dis. 2025 Jun 3;19(6):e0013127. doi: 10.1371/journal.pntd.0013127 (PMC12157779; doi:10.1371/journal.pntd.0013127)
Supplement: Attachment — Submitted filename: Response to Reviewers.pdf [file pntd.0013127.s006.pdf]

PNTD-D-24-01586
Development of a novel EV-A71 monoclonal antibody for monitoring vaccine potency
PLOS Neglected Tropical Diseases

Response Letter

Results

- Does the analysis presented match the analysis plan?
- Are the results clearly and completely presented?
- Are the figures (Tables, Images) of sufficient quality for clarity?

Reviewer #1: Yes

Reviewer #2:

1. Figure 1 could include images of mouse inoculation to make the article more vivid and intuitive.

Author's response: Thank you for your suggestion. We have revised Figure 1 in accordance with your feedback. Please refer to Figure 1A in the updated version for further information.

2. In the potency assays section, results under UV exposure conditions should also be added.

Author's response: Thank you for your comment. Please refer to Figures 6A and 6D in the revised version for further details. The in vitro and in vivo results indicate that the antigen exposed to UV-C is an effective antigen, as it can be recognized by NHRI2016-1 and MAB979 in dot blot and induces neutralizing titers similar to those observed at 30°C and 4°C (Figure 6D).

Reviewer #3: The authors developed a monoclonal antibody (NHRI2016-1) that might be used to evaluate the potency of EV-A71 vaccines either in vitro or in vivo. They clearly showed processes of the generation, the binding site identification and the evaluation of NHRI2016-1. This is a great work. However, there are some issues need to be addressed.

Major comments:

1. How was the B5-141 that was employed to generate monoclonal antibodies inactivated? If it was inactivated by heating, it would be identified by NHRI2016-1 after heating in Result III.

Author's response: Thank you for your comment. B5-141 was inactivated with 0.02% formaldehyde for 72 hours at 37°C and used to immunize mice for monoclonal antibody generation. According to our dot blot data, NHRI2016-1 was unable to recognize EV-A71 antigens heated to extreme temperatures (90°C and 60°C), as shown in Figure 6A of the revised version.

2. Please provide additional experimental data on the in vivo effectiveness of the vaccine at various temperatures (UVed, 4°C, 30°C, 60°C and 90°C).

Author's response: We have performed the experiments following the reviewer's suggestion. Please refer to Figure 6D in the revised version for further details. The results showed that effective antigen (those exposed to 30°C, UV-C for 1 h, or kept at 4°C) could induce immune responses in rats, while inffective antigen (those heated at 90°C or 60°C for 1 h) could not.

3. Vaccines are rarely kept at 90°C in the real world. However, NHRI2016-1 can only distinguish between effective and ineffective vaccines at 90°C. Please give the practical significance of this result.

Author's response: Thank you for your comment**.** It is true that vaccines are rarely exposed to high temperatures, such as 60°C and 90°C. However, exposing vaccines to extreme temperatures, like 60°C and 90°C for 1 hour, is a common practice to assess vaccine potency across a range of conditions within a short period and to evaluate their stability and effectiveness in both in vitro and in vivo tests (doi:10.1371/journal.pntd.0002538, https://doi.org/10.1128/msphere.00088-22**)**. In addition, subjecting vaccines to extreme conditions, such as 60°C and 90°C, serves as a stress test (based on WHO guidelines on stability evaluation of vaccines, https://doi.org/10.1016/j.biologicals.2009.08.017) designed to simulate the impact of accidental temperature fluctuations that may occur during transportation or improper storage. This test provides valuable insights into the vaccine's stability and the possible degradation of its antigenic components under unfavorable conditions.

In Figure 6A, NHRI2016-1 distinguishes between effective vaccines (30°C heated, 4°C unheated, and UV-C-exposed) and ineffective vaccines (90°C and 60°C heated), reflecting similar results observed in in vivo testing (Figure 6D). The practical significance of this result lies in the ability to assess the robustness and resilience of vaccines. By using NHRI2016-1 to evaluate vaccine performance under these stress conditions, manufactures can gain a better understanding how their products might behave when exposed to high temperatures, even if such temperatures are unlikely to be encountered during normal storage. This type of testing can inform the development of more stable

vaccines, improve quality control processes, and ultimately ensure that vaccines maintain their potency, safety, and effectiveness, even in less-than-ideal storage or transport environments.

We have included the explaination for using these extreme temperatures in the discussion section, please refer to lines 624-646 in the revised version for further information.

Minor comments:
1. Line 44. Please give the full name of HFMD.

Author's response: Please refer to lines 45-46 in the revised version for further details. HFMD stands for hand, foot, and mouth disease.

2. Line 80. It is recommended to add the reason that MAB979 failing to distinguish between effective and ineffective vaccines.

Author's response: Thank you for your suggestion. Based on the dot blot assay and rat immunization study, we found that MAB979 was unable to distinguish between effective and ineffective vaccines in this study. Since MAB979 recognizes linear epitope formed by VP2 residues 141-150 (10.1016/j.vaccine.2011.04.010), it is possible that this epitope remains exposed and intact in both effective and ineffective vaccines. This could explain why MAB979 is unable to differentiate between them. We have included this explaination in the discussion section, please refer to lines 566-571 for more details.

3. Line 163. Please show the sequence or reference of specific primers.

Author's response: Thank you for your comment. For further details, please refer to S1 Table in the revised version, which provides additional information about primer sequences.

4. Line 277. Why don't the authors evaluate the genotypes D, E, F and G?

Author's response: Thank you for your comment. The EV-A71 genogroups E and F were identified in Africa, while genogroups D and G were first isolated in India. Currently, we do not have these viruses in our lab, so we are unable to perform a neutralization assay for NHRI2016-1 against these genogroups.

5. Table 1. It is recommended to use a bar chart to display the results of this part, which is more intuitive

Author's response: Thank you for your suggestion. We have made the necessary modifications. Please refer to Figure 1C in the revised version for further details**.**

6. Line 271. Please add the results of screening to Supplementary.

Author's response: Thank you for your comment. We have added the screening results to Figure 1. Please refer to Figure 1B in the revised version for further details.

7. Line 303. It is recommended to add the sequence of B5-Mut3-1 to GenBank.

Author's response: Thank you for your suggestion. The GenBank accession number for B5-Mut3-1 is PV176308. It will be released in the NCBI database on August 28, 2025. The image below shows the email response from NCBI GenBank.

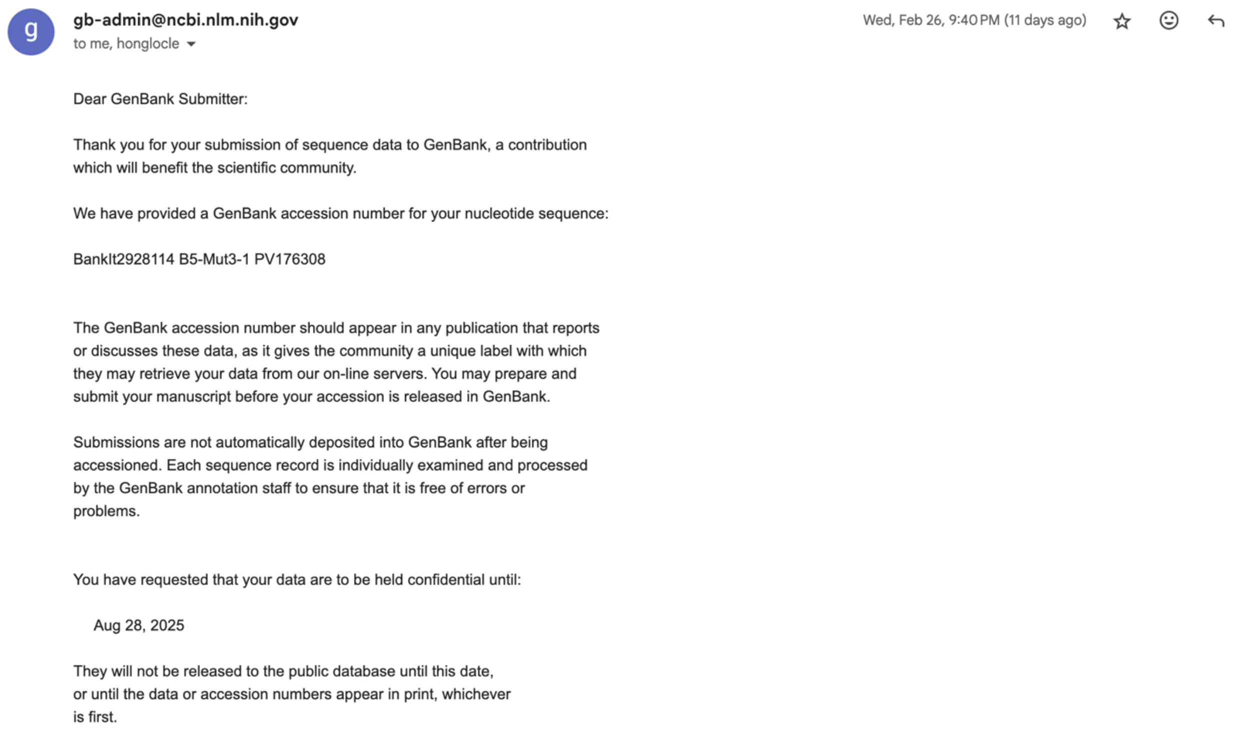
gb-admin@ncbi.nlm.nih.gov

to me, honglocle

Wed, Feb 26, 9:40 PM (11 days ago)

Dear GenBank Submitter:

Thank you for your submission of sequence data to GenBank, a contribution which will benefit the scientific community.

We have provided a GenBank accession number for your nucleotide sequence:

BankIt2928114 B5-Mut3-1 PV176308

The GenBank accession number should appear in any publication that reports or discusses these data, as it gives the community a unique label with which they may retrieve your data from our on-line servers. You may prepare and submit your manuscript before your accession is released in GenBank.

Submissions are not automatically deposited into GenBank after being accessioned. Each sequence record is individually examined and processed by the GenBank annotation staff to ensure that it is free of errors or problems.

You have requested that your data are to be held confidential until:

Aug 28, 2025

They will not be released to the public database until this date, or until the data or accession numbers appear in print, whichever is first.

8. Can UV render ineffective vaccines undetectable by NHRI2016-1?

Author's response: EVA71 B5 particles were exposed to UV-C light (wavelength of 253.7 nm, a typical UV germicidal lamp found in biosafety cabinet type 2 [G30T8, Sankyo Denki, Japan]) for 1 hour. A dot blot assay was then performed to detect the signal using either NHRI2016-1 or MAB979 (Figure 6A). The results demonstrated that NHRI2016-1

successfully detected the UV-C exposed sample. Additionally, the UV-C exposed sample was able to induce neutralizing antibodies, with results comparable to those observed at 30ºC and 4ºC (Figure 6D). Therefore, these samples are still considered effective vaccines.

The UV-C spectrum (200-280 nm) is widely recognized as the germicidal range for UV irradiation. Within this range, viral nucleic acids (including RNA and DNA) can absorb UV-C photons, leading to significant damage. The absorbed photons induce crosslinking between pyrimidine bases in the viral RNA, forming dimers that hinder proper replication or translation. Therefore, the effect of UV irradiation is referred to as "inactivation" rather than "killing" (https://pubs.acs.org/doi/epdf/10.1021/acsphotonics.0c01245?ref=article_openPDF). Since our vaccine is an inactivated whole virion vaccine, UV-C irradiation does not destroy the structure of the EV-A71 virion, as observed in TEM analysis (Figure 6B). Previous studies have also suggested that UV irradiation insignificantly contributes to capsid damage in both enveloped and non-enveloped viruses (https://doi.org/10.1039/d3ew00277b, https://doi.org/10.1038/s41598-021-93231-7). However, we believe UV-C may have a detrimental effect on DNA, RNA, or live attenuated vaccines.

Reviewer #4: (No Response)

Summary and General Comments

Use this section to provide overall comments, discuss strengths/weaknesses of the study, novelty, significance, general execution and scholarship. You may also include additional comments for the author, including concerns about dual publication, research ethics, or publication ethics. If requesting major revision, please articulate the new experiments that are needed.

Reviewer #1:

1. Abstract: It might be worth including that EV-A71 causes FMHD in the abstract. Its included in the next section (author summary), but I recommend it including it in the abstract for the benefit of the reader.

Author's response: Thank you for your suggestion. We have made revisions based on your suggestion. For further details, please refer to lines 14-15 in the revised version.

2. Introduction: Lines 89-91: "We also identified the binding epitope of NHRI2016-1, which in turn may have important implications for EV-A71 vaccine development". Based on the

experiments and the results, it seems that one conserved amino acid on the antigen (VP3-S64) was identified to be key in binding of the mAb to the antigen. Is it fair to claim that you have identified the "epitope", when multiple amino acids located in different regions come together to form the conformational epitope that would potentially be involved in the binding of the mAb to the antigen? You might have to employ CryoEM to get a complete picture of the multiple amino acids involved to form the epitope. Please clarify.

Author's response: Thank you for your suggestion. You are correct that the term "epitope" refers to the part of an antigen recognized by an antibody, and this epitope can consist of multiple amino acids located in different regions of the antigen. In our study, we identified VP3-S64R as a key amino acid involved in the binding of NHRI2016-1 to the antigen. However, we acknowledge that there may be additional residues involved in the overall binding interaction. While we do not have access to techniques like CryoEM, which could provide a more complete structural view, our current findings suggest that VP3-S64R plays a pivotal role in the binding interaction. Therefore, although we refer to VP3-S64R as part of the binding site, further structural studies are necessary to fully define the complete binding site and the precise conformational arrangement of amino acids involved in the binding of NHRI2016-1 to the antigen.

Based on your feedback, we would like to revise this section as follows: "We identified VP3-S64R as a key amino acid involved in the binding site of NHRI2016-1, which in turn may have significant implications for the development of EV-A71 vaccines". Please refer to lines 106-108 in the revised version for further details. We have also included this part in the discussion section, please refer to lines 621-624 in the revised version for further information.

3. Neutralization Assay (Methods section) and Table 1 data: How was the cross reactivity evaluated. In the methods section, the authors mention that HG-B5-141-6-5 which is the B5 genotype. What about the others?

Author's response: Thank you for your comment. The cross-reactivity of NHRI2016-1 and MAB979 against A/BrCr, B4-E59, B5-2008, and C4-2008 was assessed using a neutralization assay. We have added more details to the methods section. Please refer to lines 295-299 in the revised version for further information. Additionally, Table 1 has been converted to Figure 1C. Please refer to lines 343-351 and Figure 1C in the revised version for more details.

4. Results section, Lines 269-273: "Given the urgent need for EV-A71-specific mAb used in in vitro potency assays, we generated hybridoma clones using isolated splenocytes from mice immunized with inactivated EV-A71 genotype B5 (B5-141). After screening by

dot-blot and neutralization assays, mAb NHRI2016-1 was selected for further expansion among five mAbs due to its strong reaction against B5-141”. A bit more background and limited key data/results regarding how this final mAb was selected would be beneficial to the readers.

Author’s response: We have added additional details and results to this section. Please refer to lines 318-320 and 337-342 in the revised version for further information.

5. Results section, lines 303-306: “To test whether B5-Mut3-1 indeed escaped from NHRI2016-1 recognition, we freeze-thawed three times prior to determining the virus titer and performing an immunofluorescence assay (IFA)”. Can you clarify what was Freeze/Thawed? Virus or the mAb?

Author’s response: We freeze-thawed the B5-Mut3-1-infected cells three times to increase viral yield before conducting virus titration and IFA. To avoid any possible confusion, we would like to revise this sentence as follows: “We then performed an immunofluorescence assay (IFA) to confirm whether B5-Mut3-1 had escaped recognition by NHRI2016-1”. Please refer to lines 379-381 in the revised version for more information.

6. Fig 3d IFA data: are the viral particles expected to be native (nondenatured) during the IFA assay. If yes, then why would MAB979 which binds to a linear epitope show a green signal with either the B5-141 or B5-Mut3-1. Is it hypothesized that the linear epitope to which MAB979 binds is on the surface of the viral particle?

Author’s response: Thank you for your comment. It is true that viral particles are preserved in their native (nondenatured) form during the IFA assay, as formaldehyde does not alter the virus’s conformation (https://doi.org/10.1038/nsmb.2255).

Linear epitopes consist of continuous amino acids sequences (polypeptides) within a protein. These sequences do not rely on the protein’s three-dimensional conformation (folding), which is why they are typically described as being recognized by antibodies in their denatured or unfolded state. However, this does not imply that linear epitopes cannot be present on native (nondenatured) viruses. As long as the linear epitopes are accessible on the virus surface, antibodies can bind to them, regardless of whether the virus is in its native or denatured form. This may explain why MAB979 is still able to recognize and bind to the linear epitope on both native (nondenatured) and denatured viruses if the epitope remains exposed and intact.

According to Liu et al. (2011) (10.1016/j.vaccine.2011.04.010), MAB979 targets the linear epitope formed by residues 141-150 of the VP2 region. We mapped these residues onto

the EV-A71 pentamer using PyMOL software, and indeed, they are located on the viral surface (please refer to image below for further details).

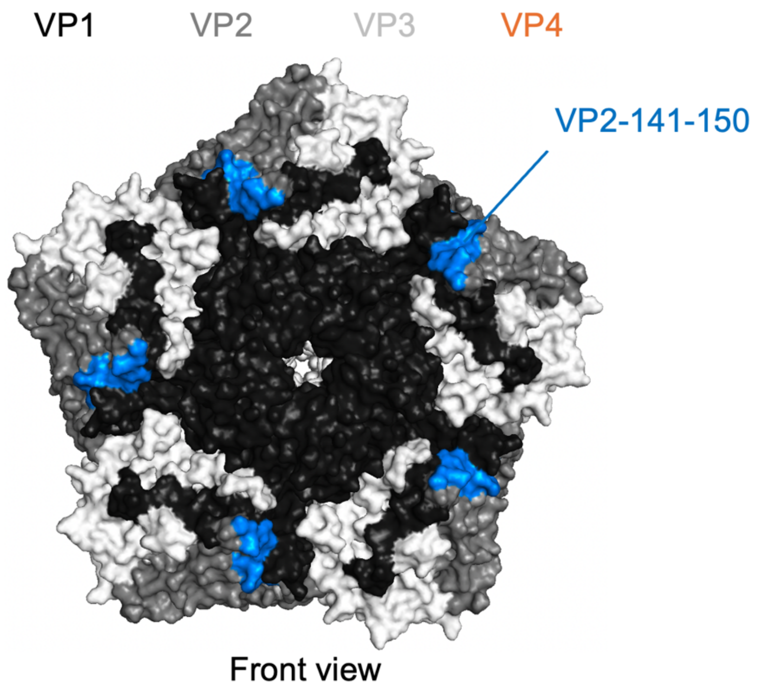

7. Table 2: raw data for the genome sequence analysis are not provided.

Author's response: Thank you for your comment. We have included the whole genome sequence analysis in S1 Fig in the revised version. Please refer to it for further details.

8. Data in Figure 3A: Does the intensity of the signal (compared to the control rgHGB5) tell us anything about the mAb binding affinity or is it just all or none phenomena as in the case of NHRI2016-1 and VP3-S64R mutant. For example, compared to the rgHGB5 control, the binding of NHRI2016-1 to the VP1, VP3-L101L, VP3Y202C is markedly different at most dilutions tested. Does lower binding in dot blot for a particular mutation indicate the role of that amino acid in the binding of the mAb to the antigen. Please comment.

Author's response: Thank you for your suggestion. We believe that the interaction follows an all-or-none phenomenon, as seen with NHRI2016-1 and VP3-S64R. The absence of signal for NHRI2016-1 binding to the VP3-S64R mutant in both dot blot and IFA suggests that the mutation may impact the ability of NHRI2016-1 to bind to the antigen. However, it is important to note that both dot blot and IFA are semi-quantitative or qualitative methods, meaning they can indicate the presence or absence of antibody-antigen interaction but cannot provide exact measurements of binding affinity. Therefore,

the intensity differences observed across various mutations (e.g., VP1, VP3-L101L, VP3-Y202C) may not necessarily correlate with changes in binding affinity.

9. Results section, Line 402-405: “First, to create a vaccine with different potency, purified EV-A71 B5 E + F particles were processed under heated (at 90°C, 60°C, 30°C) or unheated (kept at 4°C) conditions, loaded onto NC membrane in dot-blot assay, and probed with either MAB979 or NHRI2016-1 (Fig 5A)”. The duration used for the high temperature exposure is not specified.

Author’s response: Thank you for your comment. One hour is the duration used for generating different potency antigens. We have added this information in the revised version; please refer to lines 468-470 for further details.

10. Related to my earlier comment regarding the location of the linear epitope to which mAb979 binds (surface exposed or buried), how do you explain the loss in binding seen for mAb979 after exposure to 90C (signal is lower compared to 4C). If the epitope is linear and exposed (which seems likely because binding is observed at 4C), the binding should potentially not change.

Author’s response: Thank you for your comment. The amino acid sequence of the linear epitope remains unchanged, regardless of whether the virus is in its native or denatured form. Therefore, MAB979 could still theoretically bind to the denatured virus if the epitope remains exposed and intact. However, extreme heat treatment at 90°C or 60°C may cause significant unfolding, alteration, or burial of the original conformation of the VP2 residues 141-150. As a result, MAB979 may not be able to bind as effectively. This explains why the signal observed in the 90°C and 60°C heated samples is much weaker compared to the 4°C sample when using MAB979. We have incorporated the explaination for this in the discussion section, please refer to lines 566-571 in the revised version for more information.

11. Figure 5b TEM data: Was aggregation (clumping) of the antigen observed in the 90°C sample?

Author’s response: No aggregation was observed in the 90°C heated sample, either by the naked eye, TEM, or DLS (dynamic light scattering). The typical size of EV-A71 particles is approximately 20-30 nanometers (nm) in diameter (https://doi.org/10.3389/fmicb.2018.00286). According to the DLS data, no aggregation occurred, as no large particles sizes (> 100 nm) were detected in any of the samples,

including those heated at 90°C, 60°C, and 30°C, the unheated 4°C sample and the UV-C exposure sample (please refer to the image below for DLS data).

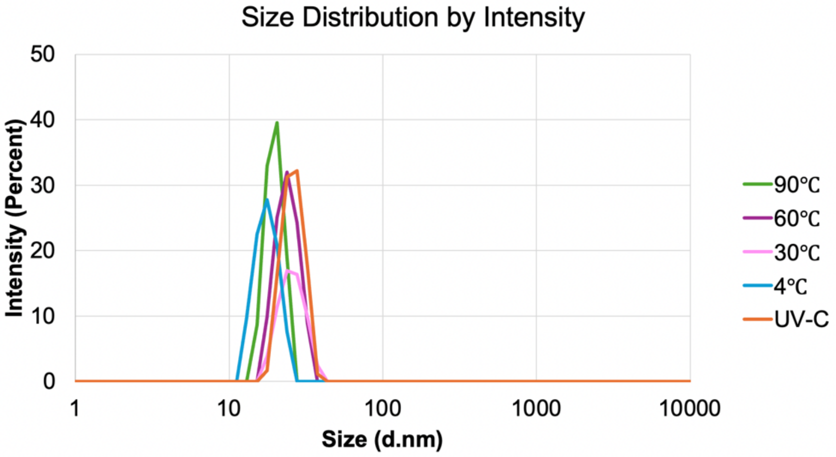

Name	Mean size (d.nm)
90°C	22.16
60°C	22.21
30°C	18.38
4°C	17.87
UV-C	21.64

12. Please comment on why 90°C incubation was deemed relevant to show in vitro in vivo correlation. Vaccines will never get exposed to such a high temperature. Instead, why not choose an extended incubation at 25 or 30°C for several months (3 or 6 months) to demonstrate the correlation under more relevant conditions that the vaccine may be exposed to during manufacturing, storage, and especially during shipment to areas where there is a lack of cold chain facilities (rural regions).

Author's response: Thank you for your comment. The decision to include 60°C and 90°C incubation was based on the goal of conducting a stress test to evaluate the stability and potency of the vaccines under extreme conditions, simulating worst-case scenarios. While it is true that vaccines are not typically exposed to such high temperatures during normal storage and transport, heat exposure at 60°C and 90°C serves to stress-test the vaccine's integrity and provide a clear benchmark for its resilience to temperature-induced degradation. This extreme condition helps assess the robustness of the vaccines and the potential impact of accidental exposure to high temperatures during transit or improper storage.

That said, we understand the importance of using realistic temperature conditions, such as extended incubation at 25°C or 30°C for several months, to demonstrate the correlation between in vitro potency and in vivo immunogenicity under more relevant storage and transport conditions. However, conducting such long-term studies may not be feasible in the short term, as several months of incubation could be a lengthy process for screening purposes.

Our current focus was to screen whether NHRI2016-1 could differentiate between effective and ineffective vaccines, and our results have shown that NHRI2016-1

successfully distinguishes between these two. Given that our primary aim was to test the antibody's ability to differentiate vaccine quality in a relatively short time frame, the extreme heat exposure provided a rapid and effective means of testing this capability. For further studies, we believe collaboration with other labs to establish international standards for vaccine stability and conduct more extensive quality control (QC) and quality assurance (QA) tests is crucial. This would allow for more realistic, long-term stability testing under practical conditions that vaccines are likely to encounter during manufacturing, storage, and shipping, especially in areas with limited cold chain facilities.

We have included explaination for using these extreme temperatures in the discussion section, please refer to lines 624-646 in the revised version for further information.

13. For the in vivo experiments, it's definitely possible that there was conformational change in the antigen after 90°C exposure and there a response was not seen after injecting in mice. However, there is a possibility that the antigen was complete aggregated and might have fallen out of solution after 90°C exposure (formation of visible particles), which would change the concentration of the antigen in solution. How did you ensure that there was no aggregation, and you injected the same amount of antigen (400 IU) into the animal for both the 90°C and the 4°C arm?

Author's response: Thank you for your suggestion. We agree that there must be a conformational change in the samples heated at extreme temperatures, but we are unable to observe the details using TEM. However, we can confirm that no visible particle formation occurred after extreme heat treatment (90ºC or 60°C), as observed both by the naked eye and through DLS analysis (please refer to the image below and the answer to question 11).

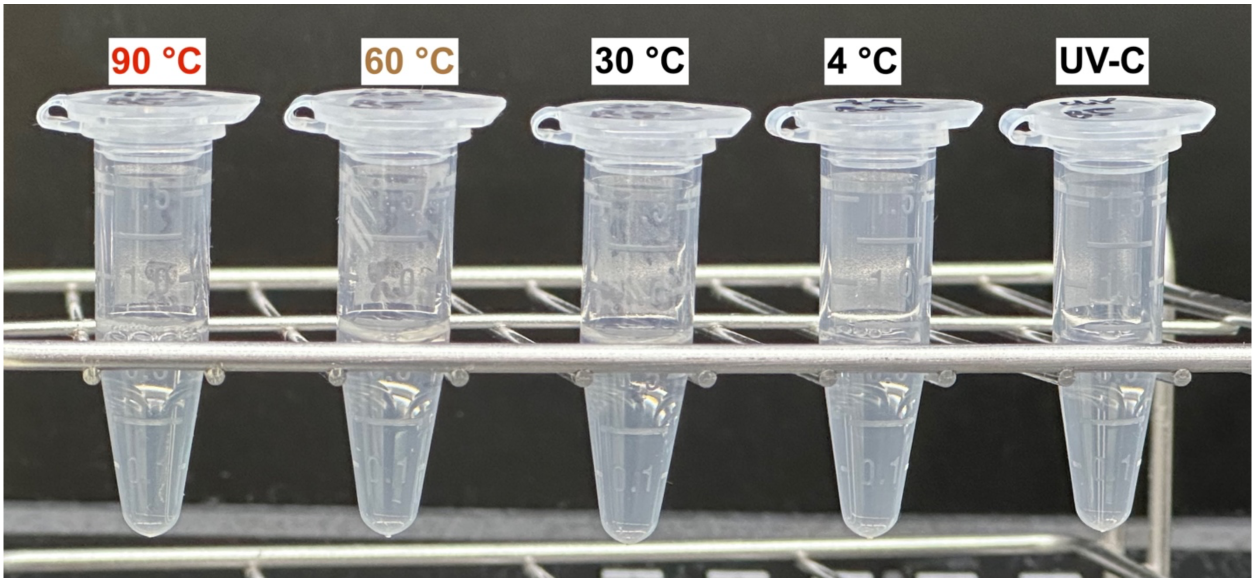

To ensure consistent antigen injection (400 IU) across the five groups, we first conducted an ELISA (please refer to lines 253-264 in the revised version for further details) on the purified B5 antigen bulk, which is always stored at 4°C. The measured ELISA unit for the antigen bulk is 7.882 IU/uL, and we need 400 IU for each dose per rat. Therefore, the volume required per rat is 400/7.882 = 50.74 uL. Since one group consists of 4 rats, and two doses are administered, we prepare antigen for 9 rats, which totals 456.7 uL per group. Additionally, we need some antigens for DLS (100 uL) and TEM (5-10uL), so we prepare a 700 uL aliquot of antigen for each group. Each aliquot is then processed under different temperature conditions or UV-C exposure. After heat, unheat, or UV-C treatment, these antigen are stored at 4°C and used for various tests.

For the immunization study, the adjuvant concentration is 5 ug/uL, and the required amount for each rat is 30 ug. Therefore, each rat requires 30/5 = 6 uL of adjuvant. The image below shows the calculation for the entire immunization study. The mixture of adjuvant, antigen, and PBS was evenly divided into 100 uL per syringe for each rat.

			per rat				4.5 rat			
Group	EVA71 (400 IU)	Adjuvant (ug)	EVA71 (ul)	Adjuvant (ul)	PBS (ul)	Total (ul)	EVA71 (ul)	Adjuvant (ul)	PBS (ul)	Total (ul)
1	B5 E + F	30	50.74	6.00	43.26	100.00	228.35	27.00	194.65	450.00
1	90oC	4 rats/group					456.70			
2	60oC						456.70			
3	30oC						456.70			
4	4oC						456.70			
5	UV						456.70			
Total		20 rats								

14. Figure 5d: If I understand correctly, the control shown in the whisker plot was an antibody used in the neutralization assay and was not an additional arm in the in vivo experiment? What sample was used in the neutralization assay with this antibody? Please provide additional context regarding the control in the main text.

Author's response: Thank you for your comment. The control shown in the plot is the positive serum control, which was always run simultaneously with the D28 serum samples. We have removed this from the plot to avoid any potential confusion for the readers. For further details, please refer to Figure 6D in the revised version.

15. Any reason why the neutralization titers of NHRI2016-1 were not compared to that of MAB797 in the in vivo experiments (Fig 5D).

Author's response: Thank you for your question. Figure 5D (please refer to Figure 6D in the revised version) shows the neutralizing antibody titers of the D28 serum from different groups. It does not pertain to MAB979 or NHRI2016-1.

16. Context regarding how the potency is currently measured in the absence of an antibody like NHRI2016-1 is missing. What is the release potency assay? If potency is currently measured using an in vivo assay due to the lack of an antibody like NHRI2016-1, provide advantages of using the mAb in a dot blot assay or ELISA in terms of time and cost saving. Additionally, consider discussing how the mAb will aid in the Replacement, reduction, and refinement efforts in vaccine potency measurements.

Author's response: Thank you for your suggestion. In the absence of an antibody like NHRI2016-1, potency is typically measured using in vivo assays, where animals are challenged with the vaccine antigen, and immune responses or survival rates are analyzed. These assays serve as surrogates for vaccine efficacy but can be time-consuming, expensive, and require significant ethical considerations.

The release potency assay currently employed may involve the following:

- In vivo assays (animal studies, lethal dose (LD50) or survival rate analysis).
- In vitro assays (ELISA, dot blot).

The use of NHRI2016-1 in dot blot assay or ELISA provides several advantages in comparison to in vivo potency assays:

- Time-saving: In vivo assays can take weeks or even months to complete, while ELISA or dot blot assay can generate results in hours or days.
- Cost-effectiveness: Animal studies require resources such as housing, feeding, and ethical oversight, significantly increasing costs. On the other hand, ELISA and dot blot assay use fewer reagents and require only laboratory personnel, thus reducing operational expenses.

Contribution of NHRI2016-1 to the 3Rs (Replacement, Reduction, and Refinement) in vaccine potency testing:

- Replacement: Reducing the need for animal models. Traditionally, vaccine potency is tested using in vivo animal models (e.g., mice, rats) by measuring immune response after vaccination. If NHRI2016-1 utilized in in vitro tests can accurately predict in vivo vaccine effectiveness, it eliminates the need for many animal experiments.
- Reduction: Minimizing the number of animals used. Even if some animal testing is still required, NHRI2016-1 employed in in vitro tests can reduce the number of animals needed by screening unstable vaccines before they reach in vivo testing. By identifying degraded or ineffective batches early, only the most stable and effective vaccines will proceed to preclinical trials, thus reducing unnecessary animal use.

- Refinement: Making animal testing more ethical and reducing distress. Some animal-based potency tests involve lethal challenge studies, where animals are exposed to a pathogen to test vaccine protection. NHRI2016-1 employed in in vitro tests can refine these tests by identifying vaccine degradation early, ensuring that only effective vaccines go to preclinical trial. This minimizes animal suffering by reducing the number exposed to potentially ineffective vaccines or avoiding the need for repeated animal testing.

We have incorporated these information in the discussion section, please refer to lines 531-539 and 574-580 in the revised version for more details.

Reviewer #2: The experimental and result sections of this article are relatively complete and well-developed, but the introduction requires improvement and further consideration. In China, EV-A71 has not been detected for a long time, while CV-A6/16/10 has become the predominant circulating strain and is associated with some cases of pediatric encephalitis. Therefore, the significance of EV-A71 presented in the introduction is relatively weak. It is recommended that the authors provide more information on the vaccination and application status of the EV-A71 vaccine.

Author's response: Thank you for your valuable feedback. We acknowledge that the introduction could be strengthened by providing more context regarding the vaccination and application status of the EV-A71 vaccine, especially given the shift in the predominant circulating strains in China. However, while CVA6, CVA16, CVA10 have become more prevalent, EV-A71 remains a predominant serotype, particularly in certain regions, and the EV-A71 vaccine continues to play a crucial role in preventing severe outcomes of hand, foot, and mouth disease (HFMD), such as neurological and cardiac complications.

As reported by Hong et al. (2022) (https://doi.org/10.1016/j.lanwpc.2021.100370), between 2013 and 2019, a total of 15,316,710 probable cases of hand, foot, mouth disease (HFMD) were reported to the China CDC surveillance system. Of these, 787,197 cases (5.1%) were laboratory confirmed, and 76,982 cases (0.5%) were classified as severe. After the launch of the EV-A71 vaccine (from 2017 to 2019), the proportion of HFMD patients infected with EV-A71 slightly decreased to 24.7%, while the proportions infected with CVA16 and other enteroviruses increased slightly to 23.7% and 51.6%, respectively. This shift in the circulating strains emphasizes the need for ongoing monitoring and vaccine development, but it does not diminish the importance of the EV-A71 vaccine in preventing severe disease outcomes.

In addition, Qiao et al. (2023) (https://doi.org/10.1016/j.jiac.2022.12.006) found that the predominant circulating serotypes of enterovirus have evolved over time. From 2007 to 2012, EV-A71 was the dominant serotype in Asia. However, after 2013, CVA6 overtook

EV-A71 as the leading serotype in terms of prevalence. In Europe, EV-A71 and CVA6 showed dynamic epidemic trends from 2009 to 2018, while in Africa, EV-A76, CVA16, and EV-A71 were the main epidemic serotypes from 2007 to 2017. In the Americas, EV-A71 remained highly prevalent from 2005 to 2019, with other strains, like CVA4, CVA5, and CVA2 showing more intermittent patterns. Despite these shifts, the ongoing circulation of EV-A71 and its potential to cause severe disease underscores the continued importance of the EV-A71 vaccine.

Although monovalent EV-A71 vaccines have been launched in China since 2016, other countries are still in the process of developing and conducting clinical trials for similar vaccines. This highlights the need for continued research and vaccine development to address evolving strains and ensure comprehensive coverage. Therefore, we believe it is essential to develop monoclonal antibodies for use in potency assays to assess the effectiveness of not only commercially available EV-A71 vaccine but also those under development, ensuring they remain effective against both EV-A71 and emerging enteroviruses.

Therefore, we have included the importance of EV-A71 vaccine development in the introduction section, please refer to lines 72-85 in the revised version for further information.

Reviewer #3: (No Response)

Reviewer #4: (No Response)

Manuscript review comments Reviewer #4:

The manuscript entitled, "Development of a novel EV-A71 monoclonal antibody for monitoring vaccine potency" reported the development of monoclonal antibody to recognize the Enterovirus A71 vaccine potency after manufacturing to till the vaccine consumption. The study is well written and can be improve by following the minor modifications as commented below.

Line 24: Mention about the type of rat used.

Author's response: Thank you for your suggestion. We used Wistar rats for the immunization study. For further details, please refer to lines 279-281 in the revised version.

Line 73-76: The following sentence can be modified into single sentence. "However, exposure to extremes of cold, heat, UV light, or insufficient excipients can cause a loss of vaccine potency, which cannot be restored [17]. In some cases, although optimal conditions are maintained, vaccine potency may still degrade gradually over time [17]."

Author's response: Thank you for your comment. We would like to revise these sentences into "However, exposure to extreme cold, heat, UV light, or insufficient excipients can cause a loss of vaccine potency that cannot be restored, and in some cases, even under optimal conditions, vaccine potency may still degrade gradually over time." Please refer to lines 90-93 in the revised version for further details.

Line 80-82: Add suitable reference.

Author's response: Thank you for your feedback. We have included the reference for it. Please refer to lines 97-99 in the revised version for further details.

Line 104: This comment shall be considered as applicable for each method and steps in the manuscript. Additional details about the instrumental setup, consumables, and specific ranges of parameters could be helpful for readers to understand the present study.

Line 115: Mention temperature + or - range for 37 C, if applicable.

Author's response: Thank you for your comment. We have added additional details on this. For further information, please refer to lines 132-133 in the revised version.

Here is the reference: https://assets.thermofisher.com/TFS-Assets/LED/manuals/forma-stericycle-co2-incubator-usermanual-7010370.pdf

Line 116: Mention pH value of VP-SFM, Gibco.

Author's response: We have included the pH value for VP-SFM (Gibco) in the revised version. Please refer to line 134 for further details.

Here is the reference:

https://assets.thermofisher.com/TFS-Assets/certificate/GRIS/COA/COA_11681020_2360185_1.pdf

Line 120: Mention the source of confirmation of 80% confluency. How author confirmed it as 80%?

Author's response: Thank you for your question. We are following the manufacturer's manual (https://www.corning.com/catalog/cls/documents/application-notes/CLS-AN-209.pdf). For example, a T150 flask (Corning) with a growth area of approximately 150 cm^2 can yield an average of 1.5 x 10^7 cells. We seed 3 x 10^6 cells per flask, and prepare

one additional flask to count the cell number. After 72 hours of incubation, the cell number in each flask reaches around 1.2 to 1.4 x 10^7 cells in total (about 80 to 90% confluency). Therefore, we will use the counted cell number to infect other flasks.

Line 122: Add the filtration flow rate and instrument used.

Author's response: We have added information about the filtration flow rate and the instruments used in the updated version. For further details, please refer to lines 138-141.

Line 124: Add the experimental parameters were employed for chromatography.

Author's response: Thank you for your comment. The experimental parameters were set according to the manufacturer's instructions, with a flow rate of 2 mL/min and 1X PBS used as the dilution buffer. Please refer to lines 155-157 in the revised version for further details.

Line 125: Specify, how did filtrate analyze for its sterility after filtration? Which sterility test was used?

Author's response: Thank you for your feedback. All procedures were always conducted in a sterile environment, specifically within a Biosafety Cabinet Class 2. A 0.22 μm filter is commonly used for sterilization, as it effectively removes biological contaminants, including bacteria, mold and yeast. Therefore, 0.22 μm filtration is often referred as 'sterile filtration'.

Line 126: Authors need to mention, what they had measured by using the kit.

Author's response: Thank you for your suggestion. We have included the information as follows: ", and the total protein content was measured using a Pierce BCA protein assay kit". For further details, please refer to lines 157-159 in the revised version.

Line 132: Mention about the mice age, gender, and weight. Add details of site of administration and quantity of dosage for immunization including antigen concentration.

Author's response: The additional information about mice has been added to the updated version as follows "A group of two-month-old, pathogen-free female BALB/c mice (n = 5, body weight 151 = 20.3 g ± 1.22 g) were immunized via intraperitoneal (i.p.) injection with formaldehyde-inactivated B5-141 emulsified in Freund's adjuvant and

boosted at two-week intervals." Please refer to lines 164-167 in the revised version for further details.

Line 133: Add details of the method employed for emulsification.

Author's response: The emulsion was prepared by repeatedly mixing the antigen and Freund's adjuvant 40 times using a glass syringe with a luer lock (TOP Surgical Manufacturing Co., Ltd.), connected to a 19G (1/2"-1.1 x 38 mm) needle. The resulting emulsion was considered successfully generated when it was thick and its droplets, when added to a saline solution, did not disperse.

Line 134: Add the details of the route of sera collection from mice.

Author's response: Thank you for your comment. Sera were collected via the submandibular vein, and we have included this information in the revised version. Please refer to lines 167-168 for further details.

Line 136: Described about the method adopted for fusion of splenic cells with the myeloma cell line.

Author's response: Thank you for your comment. We have added reference for this method. For further information, please refer to line 170 in the revised version.

Line 137: Add details about the screening method.

Author's response: Thank you for your suggestion. We have included additional details about the screening method. Please refer to lines 291-295 and 302-310 in the revised version for further information.

Line 139: Add details about the incubator used.

Author's response: Thank you for your comment. We used Thermo Scientific Steri-Cycle CO_2 Incubator (Model 370) for cell and virus culture (please refer to line 132 in the revised version for further details).

Here is the incubator manual: https://assets.thermofisher.com/TFS-Assets/LED/manuals/forma-stericycle-co2-incubator-usermanual-7010370.pdf

Line 140: Specify the temperature value instead of the room temperature.

Author's response: Thank you for your feedback. We have specified the temperature instead of using room temperature. The revision is as follows: "25°C ± 1°C for 1 h". Please refer to line 174 in the revised version for further details.

Line 143: Specify the temperatures used for freeze-thaw.

Author's response: The temperature used for the freeze-thawing cycles as follows: "freezing at –80°C for 30 min, followed by thawing at 25°C ± 1°C for 15 min".

Line 152: Add short description of protocol.

Author's response: Thank you for your suggestion. We have included short description for western blot protocol. Please refer to lines 186-188 in the revised version for further details.

Line 191: Specify the duration in hours instead of overnight.

Author's response: Thank you for your comment. We have revised the duration to 24 hours instead of using "overnight". Please refer to line 227 in the revised version for further details.

Line 194: Specify the duration in hours instead of days.

Author's response: Thank you for your suggestion. We have specified the duration in hours instead of using days. For additional information, please refer to line 230 in the revised version.

Line 198: Elaborate the method used to determine inactivation of cells.

Author's response: Thank you for your feedback. We have included additional details about the inactivation process. Please refer to lines 142-153 in the revised version for more information.

Line 199: Specify the incubation temperature. How does it was determined that the three washes were able to remove formaldehyde and unbound viruses.

Author's response: Thank you for your suggestion. We have specified the incubation temperature to 37°C ± 0.3°C. For further information, please refer to lines 233-234 in the revised version. In addition, we did not validate whether three washes were sufficient to remove formadehyde and unbound viruses, but we speculate that most of them were removed and did not affect the following steps. We would like to revise this sentence as follows: "The infected cells were then inactivated with 4% (v/v) formaldehyde at 37°C ± 0.3°C for 30 min, followed by three washes with PBS.". Please refer to lines 234-236 for further details.

Line 240: Add the details of rats used, like their gender, age, and weight.

Author's response: Thank you for your comment. Further information about the rats has been added to the updated version as follows "Seven-week-old, pathogen-free female Wistar rats (body weight = 156 g ± 8.0 g) were used for the immunogenicity assay and were housed in an AAALAC-certified animal facility at the National Health Research Institutes." Please refer to lines 279-283 in the revised version for more details.

Line 455: Described in percentage reduction.

Author's response: We have included the percentage of reduction in the revised version as follows: "Following the launch of these vaccines, the proportion of severe and fatal HFMD cases in China has decreased sharply by 62.20% and 83.78%, respectively". Please refer to lines 524-526 in the updated version for further details.

Authors needs to be carefully reviewed and address the comments made before submission of the revised manuscript.
