# Supplementary material for: Development of a novel EV-A71 monoclonal antibody for monitoring vaccine potency
Source: PLoS Negl Trop Dis. 2025 Jun 3;19(6):e0013127. doi: 10.1371/journal.pntd.0013127 (PMC12157779; doi:10.1371/journal.pntd.0013127)
Supplement: Attachment — Submitted filename: Manuscript review comments.docx [file pntd.0013127.s004.docx]

The manuscript entitled, “Development of a novel EV-A71 monoclonal antibody for monitoring vaccine potency” reported the development of monoclonal antibody to recognize the Enterovirus A71 vaccine potency after manufacturing to till the vaccine consumption. The study is well written and can be improve by following the minor modifications as commented below.

Line 24: Mention about the type of rat used.

Line 73-76: The following sentence can be modified into single sentence. “However, exposure to extremes of cold, heat, UV light, or insufficient excipients can cause a loss of vaccine potency, which cannot be restored [17]. In some cases, although optimal conditions are maintained, vaccine potency may still degrade gradually over time [17].”

Line 80-82: Add suitable reference.

Line 104: This comment shall be considered as applicable for each method and steps in the manuscript. Additional details about the instrumental setup, consumables, and specific ranges of parameters could be helpful for readers to understand the present study.

Line 115: Mention temperature + or - range for 37 C, if applicable.

Line 116: Mention pH value of VP-SFM, Gibco.

Line 120: Mention the source of confirmation of 80% confluency. How author confirmed it as 80%?

Line 122: Add the filtration flow rate and instrument used.

Line 124: Add the experimental parameters were employed for chromatography.

Line 125: Specify, how did filtrate analyze for its sterility after filtration? Which sterility test was used?

Line 126: Authors need to mention, what they had measured by using the kit.

Line 132: Mention about the mice age, gender, and weight. Add details of site of administration and quantity of dosage for immunization including antigen concentration.

Line 133: Add details of the method employed for emulsification.

Line 134: Add the details of the route of sera collection from mice.

Line 136: Described about the method adopted for fusion of splenic cells with the myeloma cell line.

Line 137: Add details about the screening method.

Line 139: Add details about the incubator used.

Line 140: Specify the temperature value instead of the room temperature.

Line 143: Specify the temperatures used for freeze-thaw.

Line 152: Add short description of protocol.

Line 191: Specify the duration in hours instead of overnight.

Line 194: Specify the duration in hours instead of days.

Line 198: Elaborate the method used to determine inactivation of cells.

Line 199: Specify the incubation temperature. How does it was determined that the three washes were able to remove formaldehyde and unbound viruses.

Line 240: Add the details of rats used, like their gender, age, and weight.

Line 455: Described in percentage reduction.

Authors needs to be carefully reviewed and address the comments made before submission of the revised manuscript.
